# Non-invasive biomonitoring of polar bear feces can be used to estimate concentrations of metals of concern in traditional food

**DOI:** 10.1371/journal.pone.0305398

**Published:** 2024-06-25

**Authors:** Kristin M. Eccles, Vincent Boutet, Marsha Branigan, Markus Dyck, Peter van Coeverden de Groot, Stephen C. Lougheed, Allison Rutter, Valérie S. Langlois

**Affiliations:** 1 Environmental Health Science and Research Bureau, Health Environments and Consumer Safety Branch, Health Canada, Ottawa, ON, Canada; 2 Institut National de la Recherche Scientifique (INRS), Québec, Canada; 3 Government of the Northwest Territories, Yellowknife, Canada; 4 Government of Nunavut, Department of Environment, Igloolik, NU, Canada; 5 Biology Department, Queen’s University, Kingston, ON, Canada; 6 School of Environmental Studies, Queen’s University, Kingston, ON, Canada; BOKU: Universitat fur Bodenkultur Wien, AUSTRIA

## Abstract

The Arctic faces increasing exposure to environmental chemicals such as metals, posing health risks to humans and wildlife. Biomonitoring of polar bears (*Ursus maritimus*) can be used to quantify chemicals in the environment and in traditional foods consumed by the Inuit. However, typically, these samples are collected through invasive or terminal methods. The biomonitoring of feces could be a useful alternative to the current metal monitoring method within the Arctic. Here, we aim to 1) quantify the relationship between concentrations of metals in the feces and tissues (muscle, liver, and fat) of polar bears using predictive modeling, 2) develop an easy-to-use conversion tool for use in community-based monitoring programs to non-invasively estimate contaminant concentrations in polar bears tissues and 3) demonstrate the application of these models by examining potential exposure risk for humans from consumption of polar bear muscle. Fecal, muscle, liver, and fat samples were harvested from 49 polar bears through a community-based monitoring program. The samples were analyzed for 32 metals. Exploratory analysis indicated that mean metal concentrations generally did not vary by age or sex, and many of the metals measured in feces were positively correlated with the internal tissue concentration. We developed predictive linear regression models between internal (muscle, liver, fat) and external (feces) metal concentrations and further explored the mercury and methylmercury relationships for utility risk screening. Using the cross-validated regression coefficients, we developed a conversion tool that contributes to the One Health approach by understanding the interrelated health of humans, wildlife, and the environment in the Arctic. The findings support using feces as a biomonitoring tool for assessing contaminants in polar bears. Further research is needed to validate the developed models for other regions in the Arctic and assess the impact of environmental weathering on fecal metal concentrations.

## Introduction

The Arctic is a sink for many environmental chemicals produced globally due to global wind patterns [1]. These higher inputs of environmental chemicals increase the potential for health risk from increased exposure for humans and wildlife living in this region. Once deposited in the environment, chemicals can then enter the food chain, where they can bioaccumulate and biomagnify [1]. Chemicals including metals, polycyclic aromatic compounds (PACs), persistent organic pollutants (POPs), polychlorinated bisphenyls (PCBs), and pesticides (e.g., chlordanes) have been detected in tissues of Arctic biota like fish, polar bears (*Ursus maritimus*), and seal [1–4]. Metals (e.g., mercury, lead, cadmium) have also been detected in the blood of Inuit, with contaminant levels often higher than those of the general Canadian population [5, 6]. While the average blood total mercury (THg) concentrations were below guidelines for screening and intervention when the Inuit Health Survey was completed in 2007–2008 [5], the ongoing accumulation and persistence of contaminants in the Arctic due to global chemical production that may an exposure risk for humans [2]. Thus, ongoing monitoring of environmental sources of contaminants is essential, given that the consumption of wildlife is important for northern peoples [7].

Inuit rely on traditional foods such as polar bears for nutritional and cultural sustenance and ecological health [8], but as apex predators, this wildlife species often have the highest body burdens of bioaccumulative contaminants [1, 4]. Further, contaminants measured in polar bears also reflect the contaminant burdens of other wildlife that both polar bears and humans eat (e.g., ringed seal [*Pusa hispida*]), thus, elevated contaminants in bear tissue may reflect elevated levels in people. Further, there are also concerns about the health of these key sentinel species that result from multi-stressor influences, many of which result from the changing Arctic climate [9, 10]. For example, the increase in Arctic temperatures is reducing the sea ice extent, which makes it more difficult for polar bears to hunt and results in decreased body condition [9]. The increase in Arctic temperature is also causing a northward range shift from certain species, resulting in a shift in local food webs and altering the flow of contaminants through the ecosystem [2]. These environmental changes can result in nutritional stress, physiological stress, and increased exposure to contaminants [2, 9, 10].

A One Health approach seeks to understand the interrelated health of humans, wildlife, and the environment, and biomonitoring tools can support this approach [11]. Biomonitoring is the measurement of chemicals in a living organism’s body fluids or tissues. The measured concentration reflects the total exposure from all routes (i.e., ingestion, inhalation, and absorption) [12]. Wildlife biomonitoring has long been used to quantify levels of contaminants in the environment and ascribe risk using key sentinel species [12–14]. While invasive or destructive samples are used, as is the case for the collection of liver or muscle samples, there have recently been initiatives to move towards non-lethal and non-invasive biomonitoring techniques, including the use of fur [15, 16] and feces [17].

Feces have promise as a biomonitoring tool, especially in the Arctic, where temperatures are below-freezing for most of the year, meaning that samples may persist for longer. Fecal samples are also relatively easy to identify on the snow-covered landscapes and can be collected non-invasively. Further, feces serve as an important excretion mechanism for eliminating contaminants from the body [18, 19]. Fecal samples from free-ranging polar bears in the Arctic have been used to assess polar bear identity and population structure [20] and diet [21] but have yet to be evaluated for their utility in estimating contaminant concentrations of tissues within the body. Previous research has successfully used other excretory pathways, such as fur, to model the relationship between measured external and internal contaminants, developing conversion factors that can be used for risk screening [15].

Here, we evaluate the relationship between concentrations of metals of concern, including arsenic, cadmium, lead, mercury, and methylmercury, in feces from the colon with those in tissues (muscle, liver, and fat) using predictive modeling. Our research seeks to evaluate the potential for field scat for non-invasive biomonitoring of body burdens of contaminants of concern in polar bears by providing an easy-to-use conversion tool to estimate internal organ metal concentrations from fecal measurements. These methods will help evaluate the potential risk to individuals who consume polar bear meat as part of a traditional diet.

## Methods

### Sample collection and composition

All samples used in this study were collected under the BearWatch program, which is a community, government, and academic partnership that aims to develop methods to non-invasively monitor polar bears across the Canadian Arctic by integrating traditional ecological knowledge with genomic [20, 22, 23] and analytical techniques [3]. Sample sets of skeletal muscle, liver, subcutaneous fat from the rump area, and intestines with feces were obtained through legal hunts of polar bears sanctioned by the Government of Nunavut (GN) and the Government of Northwest Territories (GNWT). No ethics approval was needed for this study, as section 14(6)(c) of the Inuvialuit Final Agreement (IFA), also known as the Western Arctic Claim Settlement Act, Inuvialuit hunters are given exclusive permission to hunt polar bears across the Western Arctic Region. Harvesting practices are regulated through a Total Allowable Harvest (quota), and hunting practices are conducted ethically. Following ethical hunting practices, the harvest occurs in the field, where the Inuit hunters are provided with the necessary materials and training for gathering organs for research purposes.

The polar bear sample sets were collected between 2016 and 2019 from six polar bear subpopulations across the Canadian Arctic, including Northern Beaufort Sea (NB) (n = 3), Southern Hudson Bay (SH) (n = 20), Western Hudson Bay (n = 1), Baffin Bay (n = 1), Foxe Basin (n = 12), and Gulf of Boothia (n = 12). The samples also came with information on the sex (male or female) and age (subadult or adult) of the bear. There were 36 adults (30 male and 6 female), 12 subadults (6 males and 6 females), and one bear of unknown age, each consisting of one muscle, liver, fat, and intestine sample. The samples were stored at −20°C until shipment to Queen’s University, Kingston, Ontario, Canada, all ‘cold chain’. Sample sets were processed in a Level 2 laboratory at Queen’s University, with subsamples of tissue and feces sent for chemical analyses in the Analytical Services Unit, accredited by the Canadian Association for Laboratory Accreditation (CALA).

### Chemical analysis

Detailed analytical and quality assurance/ quality control (QA/QC) methods are provided in Boutet et al. (2023). In brief, the Analytical Services Unit (ASU) at Queen’s University, Kingston, Ontario, analyzed the samples for metals and elements in the tissues of the polar bears using an Agilent 7700X Inductively Couple Plasma (ICP) Mass spectrometer (MS) (Santa Clara, California, USA) and ICP- optical emission spectrometry (OES) (Varian Vista axial ICP-OES). Total Hg (THg) was quantified using cold vapor atomic absorption spectrophotometry (Milestone DMA-80 Direct Mercury Analyzer). The methylmercury (MeHg) analysis was quantified in the laboratory of Dr. Marc Amyot, University of Montreal, Montreal, Quebec, using a Tekran® 2700 Methyl Mercury Auto-Analysis System. Appropriate QA/QC methods, including sample blanks, standard reference material, and replicate measurements, were used. Concentrations are reported in μg/g dry weight (dw).

### Statistical analysis

Descriptive statistics, t-tests, correlations, and linear regression analysis use available data (non-imputed), for which only one measurement was missing for MeHg. Values below the detection limit (BDL) were replaced with ½ the detection limit, and all values were transformed to improve normality (log10). Metals and tissues that had high percentages of BDL values ≥60% were excluded from the predictive models (see Helsel (2006) [24]). All metals were detected in assayed fecal samples, with the highest percent missing being 34.7%. Based on this criterion, seven metals were eliminated entirely from the analysis: antimony, boron, tin, beryllium, chromium, aluminum, and thallium. The highest frequency of BDL values ≥60% was observed in fat samples, where 11 metals were further excluded from the analysis, including barium (Ba), cobalt (Co), lead (Pb), manganese (Mn), molybdenum (Mo), nickel (Ni), silver (Ag), strontium (Sr), thallium (Tl), uranium (U), and vanadium (V).

We tested for differences in the measured tissue metal or element concentrations between age classes (subadult or adult) or sexes (female or male) using t-tests. The reported difference uses the first group as the category that occurs first alphabetically, which is important to determine which group has a lower mean concentration. Due to the unequal sample sizes for compared groups (age: adult = 36, subadult = 12, unknown = 1, and sex: female = 13, male = 36), we used Welch’s two-sample t-tests, which assume unequal variances. The p-value was adjusted for multiple comparisons using the Bonferroni method (n comparison = 25).

When developing the predictive models, we used a real intercept due to the time-integrated nature of chemical ADME (absorption, distribution, metabolism, excretion; for more details, see Doogue and Polasek (2013) [25]). There are scenarios where internal concentrations of a contaminant may be detectable but not yet present in the feces because excretion is the last metabolic step. Thus, an intercept of zero is not biologically valid. We did not include any additional covariates, even when differences were observed between age and sex groups, as such information on sex and age may not be readily available if fecal (scat) samples are being collected from the landscape. The regressions are log-log models, and beta coefficients (estimate) are therefore interpreted as percentage change. The p-values, root mean square errors (RMSE), R-squared estimates, and mean absolute errors (MAE) are based on results from leave-one-out cross-validations (LOOCV) from the caret package in R [26] and were used to evaluate the quality of the model. While no R-squared cut-off was applied, models were deemed poor if the p-value for the estimate was >0.05 (i.e., not statistically different from zero). We only complete pairwise predictive models (e.g., As in Feces ~ As in muscle, liver, and fat) and further investigate the use of Hg and MeHg in feces using predictive models to convert the total Hg concentrations measured in the feces to MeHg concentrations in the three tissues and feces. When developing the conversion tool, we removed all relationships with low predictive power and variables with a high number of BDL values. In this tool, we only include models where the regression coefficients were significant (p < 0.05). The goal of this conversion tool is to be conservative (i.e., overestimate), thus, we use the upper 95% confidence interval of the beta coeffect to calculate the estimated tissue concentration.

### Human health risk screening

We demonstrate how these models could be used for human health risk screening using the example of MeHg. To do this, we used rates of consumption of polar bear meat from the Inuit Health Survey (IHS) food frequency questionnaire (FFQ) conducted in 2007–2008. On average, Inuit consume polar bear meat at an average rate of 9.7 ± 68 g/week [27]. We used this mean and standard deviation within a truncated normal (min = 0) bootstrapping (n = 1000) framework to simulate the population consumption distribution. Using these simulated populations, we applied the measured upper 95% confidence limit of the mean for MeHg in polar bear muscle to estimate the mercury intake (ug/week). We then repeated this process with the upper 95% confidence limit of the estimated muscle MeHg mean. To get the estimated tissue mean, we used the regression intercept and the regression coefficient’s upper 95% confidence limit to predict the muscle MeHg concentration. We assess our predicted consumption values by comparing the estimated and measured distributions and various reference values.

## Results

Metals and elements measured in polar bear feces used in the analysis are summarized in Table 1. Overall essential elements (e.g., Ca, K, P) are measured in higher concentrations than metals with no biological function (e.g., MeHg, Hg, As). On average, the ratio of MeHg to THg measured in the feces was 19.6% (95% CI = 14.4–24.8%). This falls within the 95% confidence interval of the ratios measured in fat 26.0% (95% CI = 19.6–32.4), is slightly higher than the ratio measured in liver 8.2% (95% CI = 6.0–10.4%), and is lower than the ratio measured in muscle 71.3% (95% CI = 66.0 76.7%). None of the concentrations measured in any tissue exceeded the consumption guidelines, which are detailed in Boutet et al. (2023).

**Table 1:** Summary statistics of metals measured in polar bear feces (μg/g dry weight).

	Fat		Feces		Liver		Muscle	
Element	Mean ± SD	Range	Mean ± SD	Range	Mean ± SD	Range	Mean ± SD	Range
**Silver (Ag)**	0.01 ± 0.005	0.01–0.03	0.28 ± 0.98	0.01–6.40	0.52 ± 0.42	0.13–2.80	0.01 ± 0.01	0.01–0.04
**Arsenic (As)**	1.00 ± 0.79	0.25–4.60	2.50 ± 3.29	0.25–20.00	1.39 ± 1.32	0.25–6.50	1.49 ± 1.55	0.25–5.20
**Barium (Ba)**	0.07 ± 0.05	0.05–0.36	1.92 ± 4.66	0.05–22.00	0.07 ± 0.09	0.05–0.68	0.07 ± 0.04	0.05–0.26
**Calcium (Ca)**	67.41 ± 103.96	10.00–750.00	8,726.94 ± 23,383.52	330.00–155,000.00	110.93 ± 29.84	67.00–260.00	191.93 ± 367.54	92.00–2,700.00
**Cadmium (Cd)**	0.04 ± 0.11	0.00–0.78	1.59 ± 2.37	0.01–15.50	2.07 ± 1.24	0.46–7.80	0.07 ± 0.05	0.01–0.25
**Cobalt (Co)**	0.01 ± 0.00	0.01–0.01	0.12 ± 0.26	0.01–1.35	0.01 ± 0.00	0.01–0.02	0.01 ± 0.00	0.01–0.02
**Copper (Cu)**	0.77 ± 0.87	0.25–4.30	11.91 ± 10.35	3.70–70.00	109.64 ± 41.52	40.00–200.00	5.39 ± 1.82	2.10–12.00
**Iron (Fe)**	10.09 ± 7.24	5.00–39.50	598.09 ± 1,011.73	40.00–5,400.00	287.08 ± 189.61	43.00–950.00	130.17 ± 23.63	76.50–190.00
**Total Mercury (THg)**	5.55 ± 23.79	0.01–147.00	2.69 ± 3.52	0.30–20.67	45.86 ± 41.89	6.05–158.33	0.59 ± 0.44	0.16–2.70
**Potassium (K)**	489.90 ± 334.94	110.00–1,800.00	11,060.20 ± 3,715.34	2,500.00–19,000.00	7,030.61 ± 750.33	5,000.00–8,300.00	12,150.00 ± 1,602.99	5,350.00–15,000.00
**Methylmercury (MeHg)**	0.03 ± 0.03	0.00–0.12	0.31 ± 0.33	0.02–1.73	2.37 ± 2.50	0.48–13.58	0.43 ± 0.33	0.10–1.53
**Magnesium (Mg)**	38.80 ± 28.95	9.80–120.00	1,908.37 ± 2,028.90	210.00–11,000.00	512.96 ± 51.66	410.00–640.00	847.04 ± 118.79	390.00–1,300.00
**Manganese (Mn)**	0.13 ± 0.07	0.10–0.45	13.58 ± 40.67	0.10–260.00	10.40 ± 2.12	6.00–16.00	0.59 ± 0.33	0.21–2.40
**Molybdenum (Mo)**	0.03 ± 0.01	0.03–0.06	0.28 ± 0.15	0.11–0.94	1.39 ± 0.33	0.87–2.30	0.04 ± 0.02	0.03–0.08
**Sodium (Na)**	795.31 ± 515.07	190.00–2,300.00	6,397.96 ± 2,767.82	2,700.00–14,000.00	2,243.88 ± 536.25	1,500.00–3,800.00	2,155.10 ± 550.78	1,400.00–4,300.00
**Nickel (Ni)**	0.05 ± 0.05	0.03–0.35	0.36 ± 0.69	0.03–4.20	0.05 ± 0.07	0.03–0.52	0.05 ± 0.06	0.03–0.30
**Phosphorous (P)**	414.49 ± 249.32	120.00–1,350.00	9,221.43 ± 15,312.11	1,200.00–110,000.00	8,991.84 ± 1,002.67	6,500.00–11,000.00	8,090.82 ± 971.02	3,200.00–9,400.00
**Lead (Pb)**	5.83 ± 39.98	0.03–280.00	449.55 ± 3,144.01	0.03–22,008.50	0.37 ± 0.55	0.05–3.50	0.18 ± 0.42	0.03–2.10
**Sulphur (S)**	577.04 ± 352.55	150.00–1,900.00	11,947.96 ± 5,538.21	5,500.00–31,000.00	6,571.43 ± 664.11	5,300.00–7,950.00	8,014.29 ± 907.09	3,500.00–9,500.00
**Selenium (Se)**	0.17 ± 0.13	0.05–0.68	2.99 ± 2.30	0.80–11.50	19.04 ± 30.29	3.05–195.00	1.61 ± 0.45	0.96–3.00
**Strontium (Sr)**	0.17 ± 0.13	0.10–0.64	36.11 ± 82.44	0.64–480.00	0.26 ± 0.22	0.10–1.50	0.35 ± 0.42	0.10–2.10
**Thallium (Tl)**	0.13 ± 0.05	0.10–0.32	4.90 ± 22.10	0.10–150.00	0.25 ± 0.06	0.10–0.38	0.27 ± 0.06	0.10–0.45
**Uranium (U)**	0.001 ± 0.0004	0.001–0.003	0.03 ± 0.08	0.001–0.58	0.001 ± 0.0004	0.00–0.00	0.001 ± 0.001	0.001–0.001
**Vanadium (V)**	0.01 ± 0.01	0.01–0.05	0.44 ± 1.18	0.01–5.90	0.18 ± 0.14	0.07–0.68	0.02 ± 0.02	0.01–0.09
**Zinc (Zn)**	5.17 ± 4.78	1.00–27.50	180.59 ± 199.49	13.00–1,100.00	171.14 ± 54.32	72.00–340.00	175.79 ± 43.90	61.00–240.00

Results of t-tests before correcting for multiple comparisons comparing sexes (male and female) and ages (subadult and adult) indicate both age and sex differences in some of the assayed contaminants in feces and muscle, liver, and fat, but not for most metals. Age differences were observed in Ag in fat (p = 0.04), Na in feces (p = 0.02), Cd in liver (p = 0.048), Hg in liver (p = 0.03), Se in liver (p = 0.024), and Pb in muscle (p = 0.04). Of the significant differences between age classes, mean values for metals/elements were higher in adults, except for Na, where the mean was higher for subadults. Sex differences were observed in uranium in feces (p = 0.04), Fe in liver (p = 0.002), Mn in liver (p = 0.02), Cu in muscle (p = 0.01), Mn in muscle (p = 0.003), and Mo in muscle (p = 0.04). Means for metals/elements where significant differences were found were higher in females than males. After correcting for multiple comparisons, only Fe in the liver remained significantly higher in females than in males (S1 and S2 Tables).

**Fig 1:**
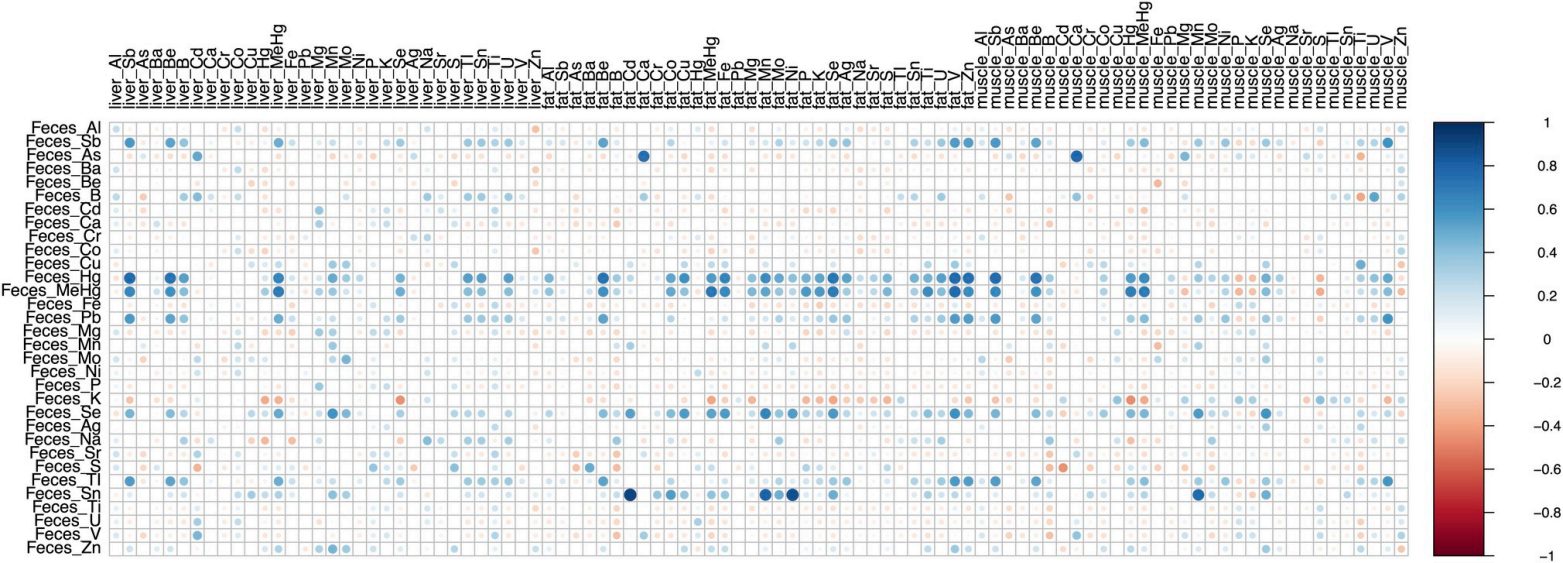
Visual representation of the correlation matrix comparing the association between each covariate. The color of the circles represents the direction of the relationship, where red indicates a negative relationship, blue indicates a positive relationship, and white represents no relation. The circle size represents the strength of the association. Cells that are missing a circle indicate non-significant correlations (p > 0.05).

Correlations imply that MeHg and Hg are broadly positively associated with various metals in various tissues (Fig 1). In contrast, some essential elements, notably P and K, were negatively associated with other tissues. The correlations between feces and fat should be interpreted with caution, as many of the metal concentrations measured in fat were BDL, as noted in the methods.

All metals were modeled in predictive linear regression models (S3 Table). The average R^2^ values range from 0.02 to 0.40. We include curated an easy-to-use conversion tool that can be used for community-based monitoring to calculate the predicted metal concentrations for any fecal metal concentration for a given tissue (S4 Table). After eliminating the metals with poor relationships (i.e., non-statistically significant beta coefficients), we were left with 16 metals in the conversion tool (As, Cd, Cu, Hg, MeHg, Pb, Mg, Mn, Mo, P, K, Se, Na, S, Ti, Zn); fourteen of these metals can be predicted in the liver, 11 in the muscle, and five in the fat.

**Fig 2:**
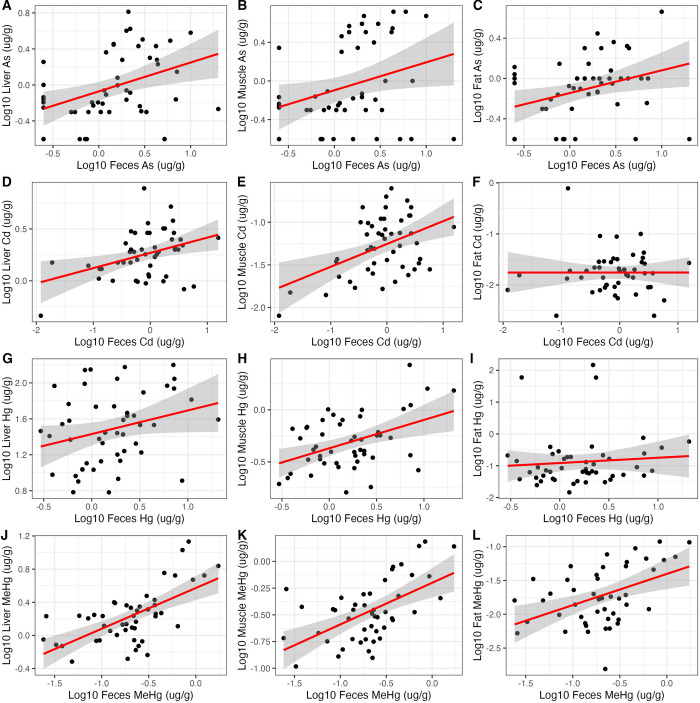
Select scatterplots with linear regression line of best fit (red) and 95% confidence interval (grey) across four different metals As (A-C), Cd (D-F), Hg (G-I), and MeHg (J-L) using the metal concentration (μg/g) measured in feces to predict the concentration of metal (μg/g) in the liver, muscle, and fat. The cross-validated regression outputs and significance corresponding to each plot can be found in Table 2.

We present the relationship between feces and internal tissues for some metals of exposure concern (Fig 2). The corresponding LOOCV regression summary statistics for the corresponding plots are presented in Table 2. When comparing As, Cd, Hg, and MeHg, the As in feces poorly predicts internal As concentration, and one average had <10% variance explained. Hg and MeHg were predictive models, as indicated by statistically significant p-values (p < 0.05) and comparatively high average R^2^ values. Fecal MeHg was more predictive of internal tissue concentration than fecal Hg; on average, fecal MeHg on average explained 40% of MeHg in the liver and 33% of variance in muscle. While Pb is also a metal of concern, many (>75%) of the concentrations measured in fat and muscle samples were BDL, thus, only liver Pb could be modeled. Pb in feces explained, on average, 27% of the variance of Pb in the liver (p < 0.001).

**Table 2:** Select leave-one-out-cross-validated (LOOCV) regression outputs using the metal concentration (μg/g) measured in feces to predict the concentration of metal (μg/g) in the liver, muscle, and fat.

Label	Tissue	Term	Beta	Error	P-value	RMSE	Average R^2^	MAE	n
**A**	Liver	Intercept	-0.07	0.05	0.18	0.36	0.09	0.3	47
		Feces As	0.32	0.11	< 0.001				
**B**	Muscle	Intercept	-0.1	0.06	0.14	0.44	0.03	0.36	47
		Feces As	0.29	0.13	0.03				
**C**	Fat	Intercept	-0.14	0.05	< 0.001	0.31	0.03	0.24	47
		Feces As	0.23	0.09	0.02				
**D**	Liver	Intercept	0.27	0.03	< 0.001	0.22	0.07	0.16	47
		Feces Cd	0.15	0.05	0.01				
**E**	Muscle	Intercept	-1.25	0.04	< 0.001	0.32	0.14	0.27	47
		Feces Cd	0.27	0.08	< 0.001				
**F**	Fat	Intercept	-1.76	0.06	< 0.001	0.43	0.68	0.3	47
		Feces Cd	0	0.1	1				
**G**	Liver	Intercept	1.43	0.06	< 0.001	0.4	0.02	0.33	47
		Feces Hg	0.26	0.14	0.06				
**H**	Muscle	Intercept	-0.37	0.04	< 0.001	0.24	0.12	0.2	47
		Feces Hg	0.27	0.08	< 0.001				
**I**	Fat	Intercept	-0.91	0.13	< 0.001	0.84	0.11	0.54	47
		Feces Hg	0.17	0.29	0.56				
**J**	Liver	Intercept	0.57	0.07	< 0.001	0.26	0.34	0.2	46
		Feces MeHg	0.49	0.09	< 0.001				
**K**	Muscle	Intercept	-0.2	0.07	0.01	0.25	0.24	0.21	45
		Feces MeHg	0.39	0.09	< 0.001				
**L**	Fat	Intercept	-1.4	0.11	< 0.001	0.39	0.15	0.33	44
		Feces MeHg	0.46	0.14	< 0.001				

**Fig 3:**
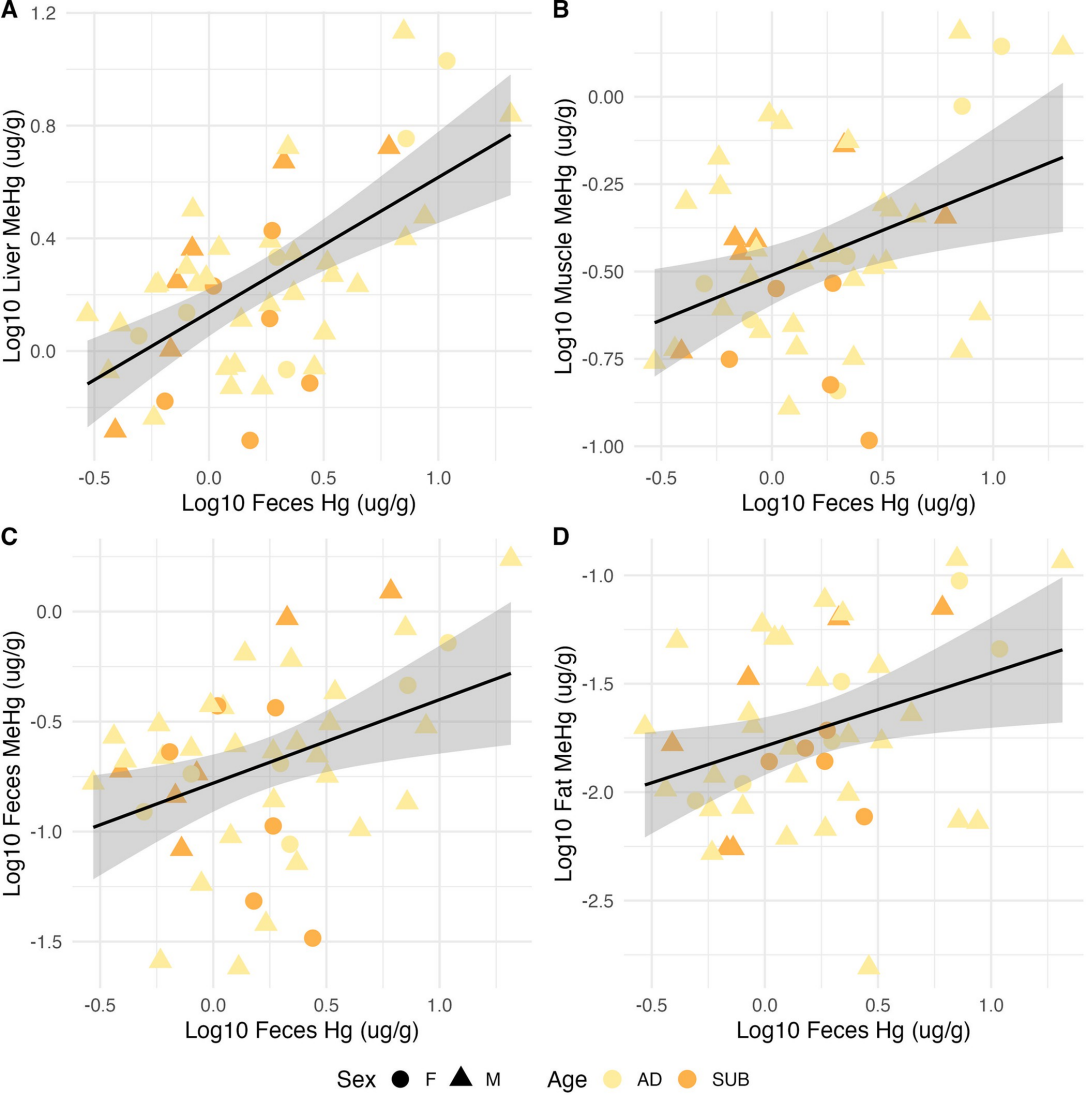
Select scatterplots with linear regression line of best fit (black) and 95% confidence interval (grey) using the Hg concentration (μg/g) measured in feces to predict the concentration of MeHg (μg/g) in the liver, muscle, and fat. The cross-validated regression outputs and significance corresponding to each plot can be found in Table 3. The sample points are also colored by age (AD = Adult, SUB = Subadult) and sex (F = Female, M = Male).

We further investigation the use of Hg and MeHg in feces as a biomonitoring and risk screening tool as it is often cheaper to measure total Hg than MeHg. We developed relationship to convert total Hg concentration in the feces into internal MeHg concentrations. There is a statistically significant relationship between the THg measured in feces and MeHg in all internal tissues, with the liver having the strongest relationship (average R^2^ = 0.37 p < 0.001) and there was no pattern by age or sex (Fig 3 and Table 3).

**Table 3:** Select leave-one-out-cross-validated (LOOCV) regression outputs using the Hg concentration (μg/g) measured in feces to predict the concentration of MeHg (μg/g) in the liver, muscle, and fat.

Label	Tissue	Term	Beta	Error	P-value	RMSE	Average R^2^	MAE	n
**A**	Liver	Intercept	0.03	0.06	0.64	0.34	0.37	0.30	47
**A**	Liver	Liver MeHg	0.79	0.15	0.00	0.34	0.37	0.30	47
**B**	Muscle	Intercept	0.47	0.11	0.00	0.41	0.16	0.35	46
**B**	Muscle	Muscle MeHg	0.56	0.20	0.01	0.41	0.16	0.35	46
**C**	Feces	Intercept	0.49	0.11	0.00	0.40	0.17	0.34	46
**C**	Feces	Feces MeHg	0.38	0.14	0.01	0.40	0.17	0.34	46
**D**	Fat	Intercept	0.78	0.25	0.00	0.42	0.15	0.34	45
**D**	Fat	Fat MeHg	0.33	0.14	0.02	0.42	0.15	0.34	45

**Fig 4:**
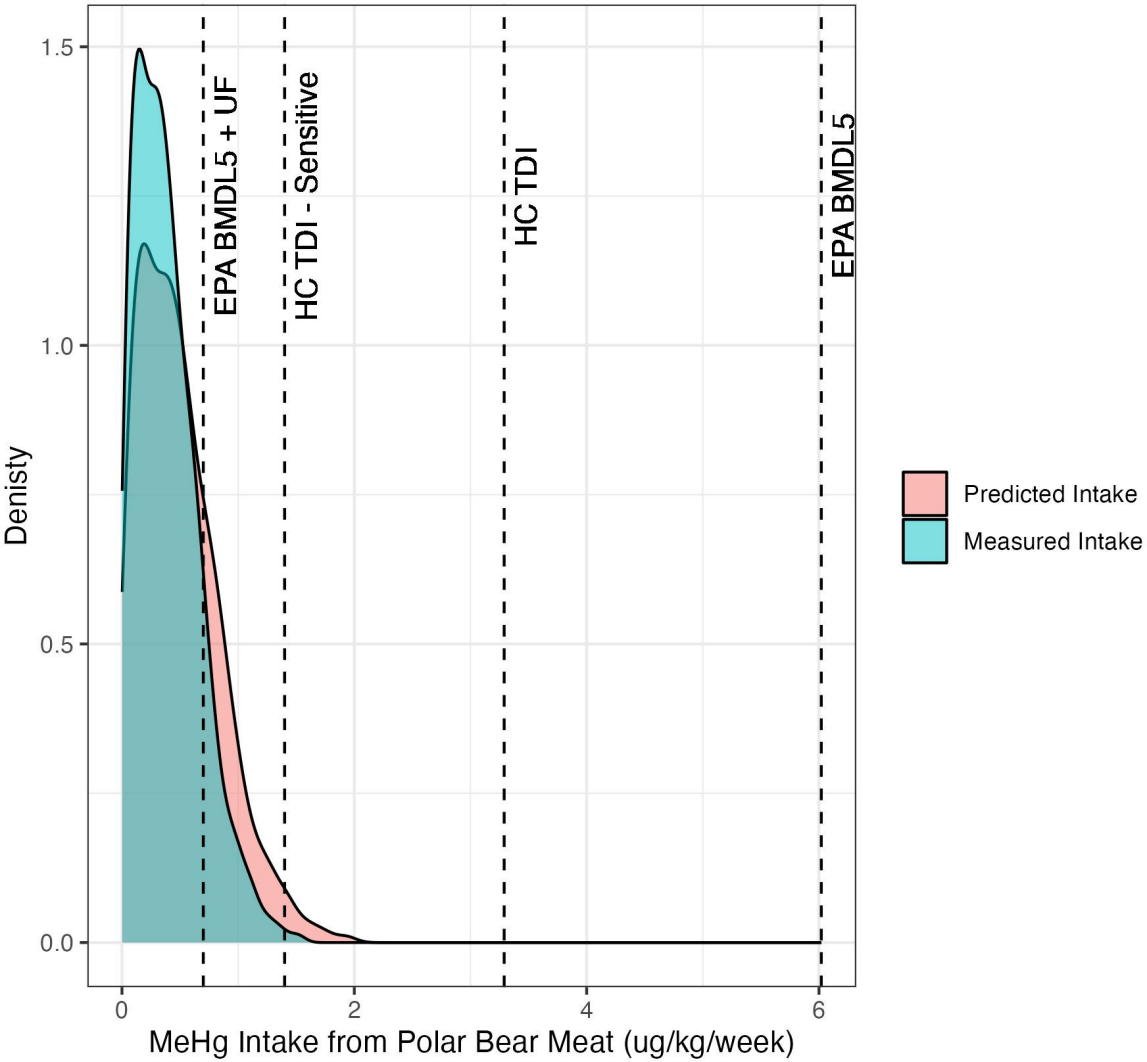
MeHg intake from polar bear meat (μg/kg/week) is based on 1000 simulations for a population using a truncated normal distribution with an average consumption rate of 9.7 ± 68 grams/week [27] of an average 67 kg person. The two histograms are the measured muscle concentration (blue bars) and muscle concentration predicted by the fecal MeHg concentration (red bars). The black dashed lines represent the varies reference values for methylmercury (MeHg).

On average, the Inuit consume 9.7 ± 68 g/week of polar bear meat [27]. Using the upper limit of the confidence interval on the beta coefficient from our models, our predictions of MeHg intake from polar bear meat is more conservative (i.e., predicts a higher concentration) for consumers on the upper end of the distribution (Fig 4 red shaded area) than the measured values (Fig 4 blue shaded area). The estimated max intake (1.96 μg MeHg/kg/week) higher than the measured intake (1.54 μg MeHg/kg/ week). This demonstrates that fecal Hg concentrations can be used to predict muscle MeHg concentrations for risk screening purposes. While the consumption rates for polar bear liver and fat were not included in Laird et al. (2013b), a similar exercise could be completed for these tissues if data becomes available.

We compare our estimated intakes to three different reference values. The provisional tolerable daily intake (TDI) for MeHg set by Health Canada is 0.0002 mg/kg/day in sensitive (infants, children <12 and women of childbearing age) and 0.00047 mg/kg/day in non-sensitive populations, which is equal to 1.4 μg MeHg/kg/ week and 3.29 μg MeHg/kg/ week, respectively [28]. The Environmental Protection Agency (EPA)’s Integrated Risk Information System assessment for MeHg lists the point of departure (POD) for oral exposure to MeHg as 8.6 x 10^−4^ mg/kg/day or 6.02 μg/kg/week based on the lower 95% confidence limit of the benchmark dose (BMDL) for developmental neuropsychological impairment. This corresponds to a reference dose (RfD) of 1 x 10^−4^ mg/kg/day (the low-end range of BMDL5) or 0.7 μg/kg/week after an uncertainty factor (UF) of 10 is applied, as this assessment is based on data from epidemiological studies [29] (Fig 4).

Based on the maximum MeHg concentration in muscle measured in our dataset and the distribution of polar bear meat consumption from average adults weighing around 67 kg [27], approximately 0.2% of the population could exceed the tolerable weekly intake using the threshold for the most sensitive population, and 12.8% of the population could exceed the EPA for MeHg with the uncertainty factor applied but are well below the empirical POD. The percentage of exceedances increases as human weight decreases.

## Discussion

Many metal concentrations measured in polar bear feces predicted contaminant concentrations in tissues; however, these relationships varied among metals and tissues with a large range of R^2^ values. Generally, concentrations measured in the feces yielded the best predictive relationships with liver, followed by muscle and fat (S1 Table). Fecal metal concentrations, particularly Hg and MeHg, had the strongest relationships with tissue concentrations. We developed a user-friendly model interface to translate the results from this study for use by community members or government officials. We use simple linear regressions rather than more complex physiologically-based toxicokinetic modeling (PBTK) as we wish to contribute to the toolbox for community-based monitoring programs and make our tools maximally accessible.

Age was missing for one female bear. While age and sex can be important, especially for bioaccumulative chemicals and chemicals that impact the endocrine system [30], there were no overarching trends of differences in metal concentrations by age, sex, or metal, especially after correcting for multiple comparisons. Not including age and sex in these models implies that the mean values of these data may change depending on the sex and age composition of future sampling (e.g., more adults than subadults). Additionally, samples from underrepresented geographic areas (e.g., Northern Beaufort Sea, Western Hudson Bay, and Baffin Bay) are needed to validate whether our developed predictive models are generalizable across all geographic regions.

Hg and MeHg models were among the best predictive models, as evidenced by the high cross-validated R^2^ values. This means that feces could be used for non-invasive biomonitoring which is especially relevant as exposure to Hg among Inuit is an ongoing public health concern [31]. This prompted our exploration of the usefulness of feces as a biomonitoring tool for human health risk screening. Total Hg measured in feces was a better predictor of MeHg concentrations in the liver than the MeHg in feces. However, total Hg in feces was not as good a predictor of MeHg in muscle but had similar predictive ability for MeHg in fat. Thus, in the absence of speciated MeHg data, total Hg could be used as a proxy for estimating internal MeHg concentrations. These results are similar, to those reported in Bechshoft et al., 2019 who use THg measured in polar bear fur as a predicter for THg and MeHg in the muscle. However, the percentage MeHg of the THg measurement was higher in the fur (> 70%) when compared with our feces (19.2%) [32]. The relationship between MeHg and Hg likely reflect some of the complex detoxification processes where MeHg is demethylated into an inorganic, and thereby, a less toxic form of Hg, or MeHg is conjugated with another compound, for example, those containing Se, which makes the MeHg inert and not harmful [33–35].

Within a One Health framework the concentration of metal measured in polar bear can also be used to draw conclusions about the ringed seals, the main prey of polar bear. Since humans and polar bears are both apex predators of seals information learned from polar bear monitoring can also be used to inform on the health of ringed seals, via contaminant burdens, and by extension humans For example, the main route of exposure to methylmercury is through the diet [36]. While the polar bear diet varies spatially, ringed sea (*Pusa hispida*) was the primary prey of polar bears in many regions in the Canadian Arctic (Baffin Bay, David Strait, Foxe Basin, Gulf of Boothia, and Southern Hudson Bay), followed by bearded seal (*Erignathus barbatus*) [37]. It is predicted that species redistribution will result from the physical and biological pressures associated with climate change [38]. A shift in available prey can also change the contaminant exposure levels and composition for wildlife through tropic level shifts [39]. Important future research would be to strengthen the understanding between the concentrations of contaminants in seals and the body burden of polar bears. Using a modeling approach based on the concentrations of contaminants in polar bear feces to estimate the concentrations of contaminants in seals could provide a low cost and non-invasive provide ongoing monitoring of ecosystem health.

Further, integration of Western scientific methodologies with traditional ecological knowledge held about the polar bear by the Inuit offers a comprehensive approach to understanding ecosystem health [40]. The conversion tool we develop in this paper can help facilitate non-invasive collection of scat from the landscape, serves as a valuable screening tool for communities to assess metal exposure in Arctic ecosystems. This holistic approach not only advances scientific understanding but also characterizes polar bear health from an Inuit perspective, acknowledging the interconnectedness of ecological, cultural, and human health in the Arctic environment [40].

Correlation plots assessing the relationship between fecal metal concentrations and the other metals in the liver, muscle, and fat could help guide further investigations of using the fecal concentration of one metal to predict the internal tissue concentration of other metals. The strong correlations, as indicated by the large red and blue circles, indicate fecal metal concentrations may predict different internal metal concentrations. This should be evaluated using larger datasets than we have here. Correlations between K and P and many tissue metal concentrations were negative (Fig 1). While we did not model these relationships using regressions, these general trends may provide additional information for communities and government agencies, as when these elements have high concentrations, it may imply lower values of some metals within tissues.

All metals assayed in this study were measured at the total concentration of all species of that element, except for MeHg. However, we know that different metal species have different bioavailability in the gastrointestinal tract and result in different toxicities [41]. For example, bioavailability for arsenic species decreases as follows: arsenite > arsenate > monomethylarsonate (MMA) > dimethylarsinate (DMA) [42]. However, in biological and environmental monitoring, measuring the total of all metal species combined is more common than quantifying speciated measures, as the latter costs more. In other words, total metal concentrations serve as a proxy exposure of the most toxic species for a given metal. In reality, this overestimates the risk from exposure but is a health-protective method that aligns with risk s guidance for evaluating metals [43]. The number of concentrations below the BDL also limited our results. While BDL values are common in toxicological research, as many environmental concentrations and exposures to chemicals are low, this can impact the modeling [24, 44, 45]. This limited the number of relationships we could derive. Moreover, while other contaminants (e.g., PACs, PCBs, and pesticides such as chlordanes) were measured in these bears [3], the large number of non-detects limited our ability to develop feces as a non-invasive biomonitoring tool for these other chemical classes. Many of these other contaminants lack toxic reference values, making it difficult to evaluate and communicate the risks of exposure.

We used feces from the lower part of the intestines, which has allowed us to develop predictive models from matched pairs between chemical concentrations in tissues and chemical concentrations in the feces of the same harvested animal. An important next step would be to test whether the conversion factors developed in this research can be applied to fecal samples (scat) collected from the landscape. While polar bears can have large home ranges (upwards of 350 000 km^2^ [46]), the fecal samples collected on the landscape can be geotagged at collection to develop regional comparisons across the different polar bear management units. Future research should test whether environmental weathering processes change the concentration of metals measured in the feces to ensure the developed relationships still represent the predicted internal concentrations.

## Supporting information

S1 Table Summary of t-test results comparing the different in measured metal concentrations by metal and tissue between male and female bears. P.adj has been adjusted for multiple comparison using the Bonferroni method.
(CSV)

S2 Table Summary of t-test results comparing the different in measured metal concentrations by metal and tissue between male and female bears. P.adj has been adjusted for multiple comparison using the Bonferroni method.
(CSV)

S3 Table Summary of the model terms for the leave one out cross validation (LOOCV) regression model using fecal metal concentrations to predict internal tissue concentrations. (CSV)

S4 Table Easy-to-use conversion tool to estimate internal tissue (yellow: muscle, liver, fat) concentrations from metal concentrations measured in feces (green). Only regression relationships where p < 0.05 are presented; black squares are where p-value regression coefficient >0.05.
(XLSX)

S5 Table Study data by tissue (Feces, liver, muscle, fat) and metal. All concentrations are reported in μg/g dry weight.
(CSV)
